# Auditory findings associated with Zika virus infection: an integrative review^☆^

**DOI:** 10.1016/j.bjorl.2019.05.002

**Published:** 2019-06-18

**Authors:** Maria Helena de Magalhães Barbosa, Maria Clara de Magalhães-Barbosa, Jaqueline Rodrigues Robaina, Arnaldo Prata-Barbosa, Marco Antonio de Melo Tavares de Lima, Antonio José Ledo Alves da Cunha

**Affiliations:** a Universidade Federal do Rio de Janeiro (UFRJ), Programa de Pós-Graduação da Faculdade de Medicina, Rio de Janeiro, RJ, Brazil; b Instituto D’Or de Pesquisa e Educação (IDOR), Rio de Janeiro, RJ, Brazil; c Universidade Federal do Rio de Janeiro (UFRJ), Maternidade-Escola, Rio de Janeiro, RJ, Brazil; d Universidade Federal do Rio de Janeiro (UFRJ), Hospital Universitário Clementino Fraga Filho (HUCFF), Departamento de Otorrinolaringologia e Oftalmologia, Rio de Janeiro, RJ, Brazil; e Universidade Federal do Rio de Janeiro (UFRJ), Faculdade de Medicina, Departamento de Pediatria, Rio de Janeiro, RJ, Brazil

**Keywords:** Zika virus infection, Hearing disorders, Hearing loss, Infecção pelo Zika vírus, Distúrbios auditivos, Perda de audição

## Abstract

**Introduction:**

Possible associations between Zika virus infection and hearing loss were observed during the epidemic in the Americas.

**Objective:**

To describe the auditory alterations, pathogenesis and recommendations for follow-up in individuals with prenatal or acquired Zika virus infection.

**Methods:**

Bibliographic research conducted in March/2018–April/2019 at the main available databases. Article selection, data extraction and quality evaluation were carried out by two independent reviewers. Studies containing auditory evaluation of patients with congenital or acquired Zika virus infection; and/or hypotheses or evidences on the pathophysiology of auditory impairment associated with Zika virus; and/or recommendations on screening and follow-up of patients with auditory impairment by Zika virus were included.

**Results:**

A total of 27 articles were selected. Sensorineural and transient hearing loss were reported in six adults with acquired Zika virus infection. Of the 962 studied children, 482 had microcephaly and 145 had diagnostic confirmation of Zika virus; 515 of the 624 children with auditory evaluation performed only screening tests with otoacoustic emissions testing and/or automated click-stimuli auditory brainstem response testing. Studies in prenatally exposed children were very heterogeneous and great variations in the frequency of altered otoacoustic emissions and automated click-stimuli auditory brainstem response occurred across the studies. Altered otoacoustic emissions varied from 0% to 75%, while altered automated click-stimuli auditory brainstem response varied from 0% to 29.2%. Sensorineural, retrocochlear or central origin impairment could not be ruled out. One study with infected mice found no microscopic damage to cochlear hair cells. Studies on the pathogenesis of auditory changes in humans are limited to hypotheses and recommendations still include points of controversy.

**Conclusion:**

The available data are still insufficient to understand the full spectrum of the involvement of the auditory organs by Zika virus, the pathogenesis of this involvement or even to confirm the causal association between auditory involvement and virus infection. The screening and follow-up recommendations still present points of controversy.

## Resumo

**Introdução:**

Possíveis associações entre a infecção pelo Zika vírus e perda auditiva foram observadas durante a epidemia nas Américas.

**Objetivo:**

Descrever as alterações auditivas, a patogênese e as recomendações de seguimento em indivíduos com infecção por Zika vírus pré-natal ou adquirida.

**Método:**

Uma pesquisa bibliográfica foi realizada em março/2018 a abril/2019 nas principais bases de dados disponíveis. A seleção dos artigos, extração de dados e avaliação de qualidade foram realizadas por dois revisores independentes. Estudos com avaliação auditiva de pacientes com infecção por Zika vírus congênita ou adquirida; e/ou hipóteses ou evidências sobre a fisiopatologia do comprometimento auditivo associado ao Zika vírus; e/ou recomendações sobre triagem e seguimento de pacientes com comprometimento auditivo pelo Zika vírus foram incluídos na pesquisa.

**Resultados:**

Um total de 27 artigos foram selecionados. Perdas auditivas neurossensorial e transitória foram relatadas em seis adultos com infecção pelo Zika vírus adquirida. Das 962 crianças estudadas, 482 apresentavam microcefalia e 145 tinham confirmação diagnóstica do Zika vírus; 515 das 624 crianças com avaliação auditiva haviam realizado apenas testes de triagem com teste de emissões otoacústicas e/ou teste de potencial evocado auditivo de tronco encefálico automático com estímulo clique. Estudos em crianças expostas no período pré-natal foram muito heterogêneos e grandes variações na frequência de emissões otoacústicas e potencial evocado auditivo de tronco encefálico automático alterados ocorreram ao longo dos estudos; alterações nas emissões otoacústicas variaram de 0% a 75%, enquanto as alterações no potencial evocado auditivo de tronco encefálico automático variaram de 0% a 29,2%. Não foi possível descartar comprometimento neurossensorial, retrococlear ou de origem central. Um estudo com camundongos infectados não encontrou dano microscópico nas células ciliadas da cóclea. Estudos sobre a patogênese das alterações auditivas em humanos estão limitados a hipóteses e recomendações ainda apresentam pontos de controvérsia.

**Conclusão:**

Os dados disponíveis ainda são insuficientes para compreender todo o espectro do envolvimento dos órgãos auditivos pelo Zika vírus, a patogênese desse envolvimento ou até mesmo para confirmar a associação causal entre o envolvimento auditivo e a infecção pelo vírus. As recomendações de triagem e seguimento ainda apresentam pontos de controvérsia.

## Introduction

The Zika virus (ZIKV) epidemic in the Americas which began in 2015, due to its significant proportions and deleterious consequences to humans, drew the attention of both the medical and lay community. Although the most emblematic fact of this epidemic was the association of ZIKV infection during pregnancy with an alarming increase in congenital microcephaly cases, other clinical manifestations were also attributed to infection by this virus,^1^, ^2^, ^3^, ^4^ such as possible associations with auditory alterations, both in acquired^5^, ^6^, ^7^ and congenital^8^, ^9^, ^10^, ^11^, ^12^, ^13^, ^14^, ^15^, ^16^ infections.

Evidence of causality between ZIKV infection and fetal abnormalities include evidence of infection during pregnancy, a specific and rare phenotype of central nervous system abnormalities in infected fetuses and newborns, and identification of the virus in the brain tissue of affected fetuses and neonates.^17^ Pioneering studies have identified a central nervous system virus tropism in infected fetuses, with devastating consequences for development, including microcephaly and other severe central nervous system malformations.^18^, ^19^ Garcez et al., through immunohistochemistry and electron microscopy using neurospheres and brain organoids, demonstrated that the Zika virus affects human neural cells, reducing their growth and viability, strongly suggesting that the virus impairs neurogenesis during human fetal development.^20^, ^21^

Considering the indications of ZIKV neurotropism and its association with malformations in affected fetuses, it may be suggested that this virus is capable of affecting auditory neural pathways or causing malformations in auditory organs, leading to their developmental impairment and, consequently, increased risk of functional or morphological auditory impairment, especially in prenatal infections. Hearing loss has been described in infections by other agents that cause congenital syndromes, such as Toxoplasmosis,^22^, ^23^ Rubella,^24^, ^25^ Cytomegalovirus,^25^, ^26^, ^27^ HIV^25^, ^28^, ^29^, ^30^ and the Herpes Virus.^25^, ^31^ Direct auditory organ injury by the virus or local inflammatory changes induced by the infection comprise the involvement mechanisms described to date.

It is known that early congenital hearing loss diagnosis and intervention facilitate the possibility of improving language and communication development prognoses in affected individuals,^32^, ^33^, ^34^, ^35^ which becomes even more important in a context of association with other malformations, limiting neuropsychomotor development, as in the case of central nervous system malformations. Thus, it is also important to better elucidate the auditory system involvement pathogenesis in both congenital and acquired infections so that preventive, therapeutic, screening or follow-up strategies can be developed.

Hearing loss, both congenital and acquired, represents an important factor that reduces the quality of life of affected individuals. Changes in auditory pathways related to ZIKV infection have been described in case reports or in small case series concerning children presenting the congenital syndrome and adults with acquired infection, but little is known about the spectrum of these changes, or their pathogeneses and prognoses. Limitations concerning knowledge on hearing impairment in infected individuals and the need for specific guidelines for early diagnosis and auditory rehabilitation make a review of the subject both relevant and necessary.

In this context, this integrative review aims to: (1) describe the functional or morphological auditory changes related to prenatal ZIKV exposure and acquired ZIKV infection; (2) describe the pathogenesis of these auditory alterations and (3) review the recommendations for hearing screening and follow-up for these individuals.

## Methods

An integrative review was performed in a systematic way according to the recommendations of the Preferred Items for Systematic Reviews and Meta-Analysis – PRISMA.^36^ The PICO strategy was used, defining Participants (P) as individuals exposed to ZIKV in the prenatal period or individuals with acquired ZIKV infection; Intervention (I) as the functional or morphological evaluation of auditory pathways, and Outcomes (O) as functional or morphological auditory system alterations.

### Literature search

The bibliographic research was carried out at the main available databases from March to April 2018 and updated until April 2019, namely PubMed/MEDLINE, LILACS, Scielo, Scopus and Web of Science, without restriction concerning language or publication date. The reference list of the selected articles, as well as Google and Google Scholar, were used to complement the search. The applied descriptors (MeSH terms) were: (Zika OR ZIKV) AND (Acoustic OR Audiometry OR Tympanometry OR Auditory OR “Evoked Potentials” OR Psychoacoustics OR “Evoked Response” OR P300 OR ABR OR BERA OR Hearing OR Hypoacusis OR Deafness OR Audition OR Dysacusis OR cochlear OR retrocochlear) (Appendix 1).

### Study selection and data extraction

Two independent reviewers, an otolaryngologist and a pediatrician with training in epidemiology, selected articles in three stages: initially by title, then by reading the abstract, and finally by reading the full text, according to pre-established inclusion and exclusion criteria. The same reviewers independently extracted data from selected articles in digital form developed for the study (Smartsheet®, Inc., Bellevue, USA). Discrepancies concerning data selection and extraction were discussed among the reviewers at the end of each step, in order to reach a consensus.

### Inclusion and exclusion criteria

All types of study design were included, as follows: (1) Description of patients with suspected or confirmed congenital ZIKV infection or patients with acquired ZIKV infection, undergoing any type of auditory evaluation; and/or (2) Hypotheses or evidence on the pathophysiology of auditory impairment associated with ZIKV infection; and/or (3) Recommendations on the screening and follow-up of patients presenting auditory impairment due to ZIKV infection.

### Quality assessment of the included studies

The quality of each study was assessed by two independent reviewers with training in epidemiology. Case report studies were evaluated from a point of view of article writing quality, using CARE (Guidelines for Case Reports) recommendations.^37^ Case series and experimental studies were evaluated for risk of bias with the instruments proposed by the NIH^38^ Items of the STROBE (Strengthening of the reporting of observational studies in epidemiology) were added to the evaluation of writing quality of the case series^39^ (Appendix 2, Appendix 3). In addition, since many studies did not focus on auditory assessments, items were added to specifically assess the quality of the report and the methodology of this assessment. Both the overall quality of the studies and the quality of the applied auditory evaluations were categorized as low, moderate or high. Discrepancies were discussed among the reviewers in order to reach a consensus.

The GRADE system was applied to assess the quality of the scientific evidence.^40^

The protocol of this review was recorded at the International Prospective Registry of Systematic Reviews (PROSPERO) under number CRD42018092819.

### Data synthesis

The data were summarized in Figures and Tables.

## Results

**Figure 1:**
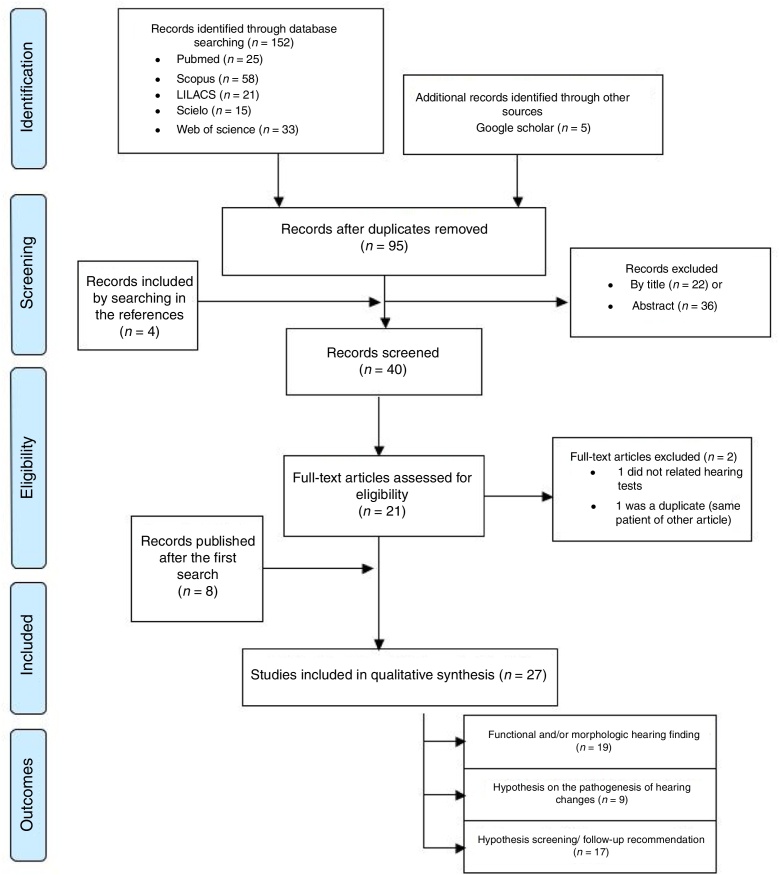
Flow diagram with the selection stages of the studies. The 27 studies included in the review were split according to the presence of each of the outcomes studied (some studies addressed more than one outcome).

The search strategy initially identified 157 records, which, at the end of the selection process, resulted in the inclusion of 27 articles^5^, ^6^, ^7^, ^8^, ^9^, ^10^, ^11^, ^12^, ^13^, ^14^, ^15^, ^16^, ^41^, ^42^, ^43^, ^44^, ^45^, ^46^, ^47^, ^48^, ^49^, ^50^, ^51^, ^52^, ^53^, ^54^, ^55^ (Fig. 1 and Appendix 1). Three of these studies concerned acquired ZIKV infection in adults (*n* = 6),^5^, ^6^, ^7^ 16 studies concerned prenatal exposure to ZIKV infection (*n* = 962)^8^, ^9^, ^10^, ^11^, ^12^, ^13^, ^14^, ^15^, ^16^, ^41^, ^42^, ^43^, ^44^, ^45^, ^50^, ^51^ nine studies included considerations on the pathogenesis of auditory impairment^6^, ^7^, ^8^, ^9^, ^42^, ^43^, ^46^, ^47^, ^52^ and 17 studies included recommendations on hearing screening and follow up for patients with congenital or acquired ZIKV infection^6^, ^7^, ^8^, ^9^, ^10^, ^11^, ^13^, ^14^, ^16^, ^42^, ^44^, ^46^, ^48^, ^49^, ^52^, ^53^, ^55^ (Table 1). Most of the nineteen studies on acquired or prenatal exposure to ZIKV were considered of low or moderate quality, both in general and in relation to the performed auditory evaluations and the quality of scientific evidence for the prevalence of hearing impairment in children prenatally exposed to ZIKV is insufficient (Table 1, Table 2). These studies comprised 968 participants, composed of six adults with acquired ZIKV infection and 962 children exposed to the ZIKV during the prenatal period.

**Table 1:** Quality evaluation of the case report or case series studies describing hearing impairment associated to Zika virus infection.

	Author, year	Title	Communication means	Study design (Type of publication)	General quality	Hearing evaluation quality
Acquired Zika infection	Tappe et al., 2015^5^	Acute Zika Virus Infection after Travel to Malaysian Borneo, September 2014	Emerging Infectious Diseases	Case report	Low	Low
	Martins et al., 2017^6^	Otological findings in patients following infection with Zika virus: case report	Audiology Communication Research	Case report	High	High
	Vinhaes et al., 2017^7^	Transient Hearing Loss in Adults Associated With Zika Virus Infection	Clinical Infectious Diseases: an official publication of the Infectious Disease Society of America	Case report	Moderate	Moderate
Congenital Zika infection	Leal et al., 2016^8^	Sensorineural hearing loss in a case of congenital Zika virus	Brazilian Journal of Otorhinolaryngology	Case report	Moderate	Moderate
	Leal et al., 2016^9^	Hearing Loss in Infants with Microcephaly and Evidence of Congenital Zika Virus Infection – Brazil, November 2015–May 2016	MMWR. Morbidity and Mortality Weekly Report	Case series	Moderate	Moderate
	Microcephaly Epidemic Research Group, 2016^50^	Microcephaly in Infants, Pernambuco State, Brazil, 2015	Emerging Infectious Diseases	Case series	Low	Low
	Van der Linden et al., 2016^51^	Description of 13 Infants Born During October 2015–January 2016 With Congenital Zika Virus Infection Without Microcephaly at Birth – Brazil	MMWR. Morbidity and Mortality Weekly Report	Case series	Moderate	Low
	Besnard et al., 2016^41^	Congenital cerebral malformations and dysfunction in fetuses and newborns following the 2013 to 2014 Zika virus epidemic in French Polynesia	Euro Surveillance	Case series	Low	Low
	Borja et al., 2017^42^	Hearing screening in children exposed to zika virus during pregnancy	Revista de Ciências Médicas e Biológicas	Case series	Low	Moderate
	Nogueira et al., 2017^12^	Adverse birth outcomes associated with Zika virus exposure during pregnancy in São José do Rio Preto, Brazil	Clinical Microbiology and Infection: the official publication of the European Society of Clinical Microbiology and Infectious Diseases	Case series	Moderate	Low
	Satterfield-Nash et al., 2017^10^	Health and Development at Age 19–24 Months of 19 Children Who Were Born with Microcephaly and Laboratory Evidence of Congenital Zika Virus Infection During the 2015 Zika Virus Outbreak – Brazil, 2017	MMWR. Morbidity and Mortality Weekly Report	Case series	Low	Low
	Silva et al., 2017^11^	Hearing screening in children exposed to Zika virus	Anals of II Brazilian Congress of Health Sciences	Case series	Low	Low
	Marques Abramov et al., 2018^43^	Auditory brainstem function in microcephaly related to Zika virus infection	Neurology American Academy of Neurology Journals	Case series	Moderate	Moderate
	Fandiño-Cárdenas et al., 2018^44^	Zika Virus Infection during Pregnancy and Sensorineural Hearing Loss among Children at 3 and 24 Months Post-Partum	Journal of Tropical Pediatrics	Case series	Low	Moderate
	Sanz Cortes et al., 2018^45^	Clinical Assessment and Brain Findings in a Cohort of Mothers, Fetuses and Infants Infected with Zika Virus	American Journal of Obstetrics and Gynecology	Case series	Moderate	Low
	Leite et al., 2018^13^	Hearing Screening in children with Congenital Zika Virus Syndrome in Fortaleza, Ceará, Brazil, 2016	Epidemiologia e Serviços de Saúde	Case series	Moderate	Moderate
	Lage et al., 2019^14^	Clinical, Neuroimaging, and Neurophysiological Findings in Children with Microcephaly Related to Congenital Zika Virus Infection	International Journal of Environmental Research and Public Health	Case series	Moderate	Low
	Gely-Rojas et al., 2018^15^	Congenital Zika Syndrome in Puerto Rico, Beyond Microcephaly, A Multiorgan Approach	Puerto Rico Health Sciences Journal	Case series	Low	Low
	Calle-Giraldo et al., 2019^16^	Outcomes of Congenital Zika Virus Infection During an Outbreak in Valle del Cauca, Colombia	The Pediatric Infectious Disease Journal	Case series	Moderate	Low
	Julander et al., 2018^54^	Consequences of in utero exposure to Zika virus in offspring of AG129 mice	Scientific Reports	Non-randomized controlled trial (in animals)	Moderate	Moderate

**Table 2:** Scientific evidence for Auditory alterations related to Zika virus according to Grading of Recommendations Assessment, Development and Evaluation.^40^

Outcomes	No. of studies	No. of patients	Frequency of altered hearing assessments	Scientific evidence	Comments
Frequency of functional auditory changes related to ZIKV exposure	15	515 (children)	OAE – 18.5%	Insufficient	Predominantly cases series
	1 case report and 14 case series	244 OAE and 448 a-ABR	a-ABR – 15.2%		Great heterogeneity of study population

OAE, Otoacoustic Emissions; a-ABR, automated Auditory Brainstem Response.

All adults presented clinical and epidemiological conditions compatible with ZIKV infection. Five were submitted for auditory evaluation exams, two of which confirmed ZIKV infection cases, while the others presented a diagnosis of probable or inconclusive infection due to possible cross-reaction with the Dengue Virus (DENV). One of the confirmed cases^5^ reported only transient hearing impairment, but the patient did not undergo any type of objective auditory testing. The hearing losses supposedly related to acquired ZIKV infection displayed a predominantly sensorineural and transient nature (Table 3).

**Table 3:** Characteristics of the studies on hearing impairment in cases of acquired Zika virus infection.

Author, year	Country of origin	Sample	Age at hearing evaluation	ZIKV and other flavivirus testing	Hearing evaluation
Tappe et al., 2015^5^	Germany	1	45 years	IgM ZIKV+ (day 6)	Not performed (hearing symptoms related)
				IgG ZIKV+ (day 6)	
				PRNT ZIKV+ (day 11)	
Martins et al., 2017^6^	Brazil	2	41 and 49 years	IgG ZIKV+	T-OAE, DP-OAE, ABR threshold-click
				IgG DENV+	Tonal and vocal audiometry with imitanciometry
Vinhaes et al., 2017^7^	Brazil	3	23, 54 and 58 years	IgM ZIKV e PRNT ZIKV+(3/3)	Audiometry
				IgM DENV+(2/3) – cross-reaction?	
				PRNT DENV+(1/3) – cross-reaction?	

ZIKV, Zika Virus; DENV, Dengue Virus; PRNT, Plaque Reduction Neutralization Test; IgG and IgM, G and M Immunoglobulins; T-OAE, Transient Otoacoustic Emissions; DP-OAE, Distortion Product Otoacoustic Emissions; ABR, Auditory Brainstem Response.

The studies concerning children presented different inclusion criteria, which can be grouped as follows: (1) Children with positive ZIKV tests with or without microcephaly at term (*n* = 102)^9^, ^10^, ^51^; (2) Children born to mothers with confirmed ZIKV infection during pregnancy (*n* = 416)^12^, ^15^, ^16^; (3) Fetus or newborns or children with suspected CZS based on epidemiologic and clinical grounds (*n* = 444)^11^, ^13^, ^14^, ^41^, ^42^, ^43^, ^44^, ^45^, ^50^ (Table 4).

**Table 4:** Characteristics of studies describing hearing evaluation in cases of congenital Zika virus infection.

Author, year	Country of origin	Total sample (*n*)	Microcephaly/neurologic changes (*n*)	Lab ZIKV positive/tested (*n*/*n*)	Age at hearing evaluation	Hearing evaluation altered/tested – (*n*/*n*) Tests performed
Leal et al., 2016^8^	Brazil	1 with microcephaly^a^	1	Mother: ND Child: 1/1 (IgM – CSF)	Newborn (age not specified)	1/1 T-OAE + a-ABR a-ABR (1 month after) + FS-ABR Behavioral hearing testing
Leal et al., 2016^9^	Brazil	70 children with microcephaly and ZIKV+ (8 preterm)	70	Mother: ND Child: 70/70 (IgM – CSF)	1st test: 114 ± 59.1 d (mean ± SD); 97 d (16–315 d) median (range) 2nd test: 1 month later	5/70 (7.1%) 1st test: a-ABR – 16/70 (22.8%) 2nd test: a-ABR – 8/16 FS-ABR (7/8; 5 with severe microcephaly and sensorineural hearing loss; 2 with conductive hearing loss)
Microcephaly Epidemic Research Group, 2016^50^	Brazil	104 children with microcephaly during the ZIKV epidemy (10 preterm)	104	Mother: ND Child: ND	NI	OEA – 2/23 (8.7%)
Van der Linden et al., 2016^51^	Brazil	13 children with ZIKV+ (2 preterm)	0 at term 11 at age 5 mo	Mother: ND Child: 13/13 (IgM – CSF/serum)	NI	a-ABR – 0/11 (0%) FS-ABR – 0/11 (0%)
Besnard et al., 2016^41^	French Polynesian	19 fetus e newborns with malformations and/or disfunctions probably caused by ZIKV (2 preterm and 11 termination of pregnancy)	8	Mother: 4/7 (PCR – amniotic fluid) Child: ND	NI	1/NI^b^ Type of evaluation not specified
Borja et al., 2017^42^	Brazil	76 children with suspected congenital ZIKV infection (8 preterm)	46	Mother: ND Child: ND	101 ± 86 d (mean ± SD)	T-OAE^c^ – 5/76 (6.6%) a-ABR – 0/5 (0%)
Nogueira et al., 2017^12^	Brazil	54 children prenatally exposed to ZIKV (8 preterm)	0	Mother: 54/54 PCR [45/53 (blood); 41/52 (urine)] Child: 18/51 PCR (14 cord blood; 4 urine)	NI	OAE – 6/34 (17.6%) a-ABR – 1/34 (2.9%)
Satterfield-Nash et al., 2017^10^	Brazil	19 children with microcephaly at term and laboratorial evidence of ZIKV infection	19^d^	Mother: ND Child: 11/19 – IgM+ PRNT+ 8/19 – PRNT+ (blood with 1–7 mo)	22 mo (19–24 mo) median (range)	13/19 (68.4%) HINE – response to auditory stimuli with rattle or bell
Silva et al., 2017^11^	Brazil	24 children with microcephaly and prenatally exposed to ZIKV	24	ND	5 mo (1–12 mo) mean and median (range)	T-OAE – 18/24 (75%) a-ABR – 7/24 (29.2%)
Marques Abramov et al., 2018^43^	Brazil	19 children with microcephaly and prenatally exposed to ZIKV	19	Mother: ND Child: 8/19 (PCR)	29.57 ± 17.84 wks (12–62 wks) mean (range)	ABR (neurodiagnostic-click) – 3/19 (15.8%)
Fandiño-Cárdenas et al., 2018^44^	Colombia	43 children born to mothers with epidemiologic criteria for ZIKV infection	3	Mother: 0/43 Child: 5/16 (PCR)	1st test: 3.5 mo (mean); 2nd test: 3 mo later 3rd test: 24 mo	OAE/a-ABR/Tympanometry – 0/43 (0%) 1st test: DP-OAE 2nd test: DP-OAE, tympanometry, a-ABR threshold-click 3rd test: DP-OAE
Sanz Cortes et al., 2018^45^	Colombia	12 children with CNS malformations exposed prenatally to ZIKV	9	Mother: 7/12 5/12 (CSF) 2/12 (placenta) Child: 1/12 (cord blood)	NI	a-ABR – 0/2 (0%)
Leite et al., 2018^13^	Brazil	45 children with CZS	45^e^	Mother: ND Child: 11/45 (type of test not specified)	10 mo (1–20 mo) mean (range)	OAE – 13/43 – 11/43 RE (25.6%) (10 altered tympanometry) and 12/43 LE (27.9%) (9 altered tympanometry) Tympanometry – 28/44 RE (63.6%) and 22/44 LE (50%) Stapedial reflex – 12/44 RE (27.3%) (10 altered tympanometry) and 11/44 LE (25%) (8 altered tympanometry) Blink reflex – 6/43 (13.9%) (5 altered tympanometry – 4 bilateral and 1 RE)
Lage et al., 2019^14^	Brazil	102 children with microcephaly and gestational ZIKV clinical symptoms	102	Mother: ND Child: ND	NI	ABR – 17.3% (*n* NI) (type of stimulus NI)
Gely-Rojas et al., 2018^15^	Puerto Rico	191 infants born to mothers with ZIKV positive test during pregnancy	15	Mother: 191/191 (PCR or IgM) Child: ND	NI	ABR – 38/191 (20%) (type of stimulus NI)
Calle-Giraldo et al., 2019^16^	Colombia	171 exposed fetuses (17) and infants (154) whose mothers developed symptomatic and confirmed Zika infection during pregnancy	7	Mother: 170/170 (PCR) Children: ND	7.6 mo ± 4.3 (mean ± SD)	ABR – 6/68 (8.8%) (type of stimulus NI)
Total		962	482	Children: 145/245 (PCR or IgM in CSF or blood or urine)		Any informed auditory evaluation – 624 OAE or a-ABR – 515^f^

d, days; mo, months; wks, weeks; CSF, Cerebrospinal Fluid; Aabr, automated Auditory Brainstem Response; NI, Not Informed; CP, Cephalic Perimeter; ND, Not Determined; IgM, Immunoglobulin M; PCR, Polymerase Chain Reaction; PRNT, Plaque Reduction Neutralization Test; T-OAE, Transient Otoacoustic Emissions; DP-OAE, Distortion Product Otoacoustic Emissions; ABR, Auditory Brainstem Response; FS-ABR, Frequency Specific Auditory Brainstem Response; HINE, Hammersmith Infant Neurologic Exam; CZS, Congenital Zika Syndrome; RE, Right Ear; LE, Left Ear.

a This case is included in the case series of the same author. However other hearing tests were performed in the follow-up of this case. This child was removed from the counting of the total number of children in the studies, since it is duplicated.

b This child with altered hearing test was microcephalic.

c Only one of the children with altered hearing test was microcephalic.

d 4 of the 19 cases had normal CP at reassessment (probable misclassification at birth).

e The median cephalic perimeter at birth was 29.4 cm (SD = 2.34).

f Including 259 children in which the type of the stimulus in the ABR was not specified (probably a-ABR).

Among the 962 fetus or children studied, 482 presented microcephaly or neurologic changes; 245 were tested for ZIKV by PCR and/or IgM determinations in CSF and/or blood and/or urine, and a total of 145 presented laboratory confirmation of prenatal infection (Table 4).

Among the 624 children with informed auditory evaluations, 515 performed a hearing screening test with transient or distortion-product otoacoustic emissions testing (T-OAE or DP-OAE), and/or automated Auditory Brainstem Response testing (a-ABR) to determine auditory threshold values. These included 259 children in which the type of ABR stimulus was not specified, but were probably a-ABR. In one study including 102 children with microcephaly, the absolute number of children that performed ABR could not be determined. Nineteen children, in addition to a hearing screening, also underwent a diagnostic evaluation by a Frequency-Specific Auditory Brainstem Response testing (FS-ABR), one of which also performed a behavioral auditory evaluation. Forty-four children underwent tympanometry in addition to OAE, 68 children performed Hammersmith Infant Neurologic Exam (HINE) in addition to ABR, while 19 underwent only HINE (Table 4).

The mean age of 296 children in the first hearing assessment was over 3 months old and not specified for the rest of the children. Most children underwent only one or two assessments at 1 month intervals. Only 43 children underwent three evaluations between 3, 6 and 24 months of age (Table 4).

Great variations in the frequency of altered OAE and a-ABR occurred across the studies: altered OAE varied from 0% to 75%, while altered a-ABR varied from 0% to 29.2% (Table 4).

Of the total number of patients who underwent OAE assessments (*n* = 244), 18.4% presented alterations, while 25% of microcephaly cases displayed alterations. Among the 448 patients who reportedly underwent the first a-ABR test (including those in which the type of ABR stimulus was not specified), 15.2% presented alterations, while in some studies data were not available to estimate the percentage of altered exams among microcephalic patients (Table 5).

**Table 5:** Absolute and relative frequency of children with altered hearing tests, overall and according to the presence of microcephaly.

Hearing evaluation	Studies	Altered tests/tests performed *n*/*n* (%)	Microcephalic/altered tests *n*/*n* (%)	Altered microcephalic/tests microcephalic *n*/*n* (%)
T-OAE or DP-OAE	Borja et al. (2017)^42^; Fandiño-Cárdenas et al. (2018)^44^; Leal et al. (2016)^8^; Microcephaly Epidemic Research Group (2016)^50^; Nogueira et al. (2017)^12^; Silva et al. (2017)^11^; Leite et al. (2018)^13^	45/244 (18.4)	35/45 (77.8)	35/140 (25)
First a-ABR	Borja et al. (2017)^42^; Calle-Giraldo et al. (2019)^16^; Fandiño-Cárdenas et al. (2018)^44^; Gely-Rojas et al. (2018)^15^; Leal et al. (2016)^9^; Nogueira et al. (2017)^12^; Sanz Cortes et al. (2018)^45^; Silva et al. (2017)^11^; Van der Linden et al. (2016)^51^	68/448^a^ (15.2)	NA/68	NA/NA
ABR (Neurodiagnostic-click)	Marques Abramov et al. (2018)^43^	3/19 (15.8)	3/19 (15.8)	3/19 (15.8)
FS-ABR	Leal et al. (2016)^9^; Van der Linden et al. (2016)^51^	5/19^a^ (26.3)	5/5 (100)	5/19 (26.3)
Behavioral Audiometry	Leal et al. (2016)^8^	1/1 (100)	1/1 (100)	1/1 (100)
Other hearing evaluations^c^	Satterfield-Nash et al. (2017)^10^; Besnard et al. (2016)^41^	14/20^b^ (70)	14/14 (100)	14/20^b^ (70)

a-ABR, automated Auditory Brainstem Response; T-OAE, Transient Otoacoustic Emissions; DP-OAE, Distortion Product Otoacoustic Emissions; ABR, Auditory Brainstem Response; FS-ABR, Frequency Specific Auditory Brainstem Response.

a 5 patients with sensorineural hearing loss and severe microcephaly (1 patient duplicated in the 2 studies of Leal et al., 2016 – removed from numerator and denominator).

b The study of Besnard et al. does not specify how many children were tested with hearing tests; only reports that one of them had altered test.

c 19 patients were submitted to HINE (Hammersmith Infant Neurologic Exam) – response to auditory stimuli with rattle or bell – and one child had the hearing test not specified.

Among the three studies that included only children with laboratory confirmation of congenital ZIKV infection (*n* = 102),^9^, ^10^, ^51^ 18 had hearing alterations (17,6%): five in the ABR and 13 in the HINE (Table 4).

The study carried out by Van der Linden et al.^51^ examined children with late onset microcephaly and did not find any alterations in the performed hearing tests. The study performed by Marques Abramov et al.^43^ was the only one to evaluate microcephalic children by neurodiagnostic click ABR, in order to evaluate auditory pathway integrity, and not auditory threshold values. The authors found only mild latency changes in three patients.

No histological studies in humans concerning auditory pathway involvement pathogenesis in ZIKV infection cases were found. The considerations of several authors on the pathogenesis are summarized in Table 6. In addition, no studies indicating morphological alterations in organs/auditory pathways related to ZIKV infection were reported. One experimental study was found addressing the auditory effects of the intrauterine exposure to ZIKV in no-human mammals.^54^ The hearing evaluation was both functional, with electrophysiological tests – ABR and Terminal Cochlear Action Potentials – and histopathological, with the microscopy of the cochlear hair cells. Auditory alterations were found in 25–66% of the mice, in the FS-ABR, depending on the frequency of the tone-burst, with a greater number observed at higher frequencies. No deficits were observed when using click stimulus. It also reported the results of the microscopic analysis of the cochlea of these offsprings where no changes in the number of hair cells were observed.

**Table 6:** Considerations on the pathogenesis of auditory impairment according to different authors.

Author (year); country	Hypothesis about the pathogenesis
Martins et al. (2017); Brazil^6^	“Hearing compatible with normal cochlear function presenting with neural synchrony changes can be classified as a neuropathy. As such, it is verified that this definition is compatible with the characteristics observed in both cases in this study”. “In view of all the information quoted and analyzed, it is suggested that ZIKV can damage auditory nerve pathways and, in that way, impair communication in adult patients”.
Vinhaes et al. (2017); Brazil^7^	“The mechanism of SNHL associated with acute virus infection involves damage of the inner ear or auditory nerve, by a direct viral effect or mediated by an autoimmune process as previously described”.
Leal et al. (2016); Brazil^9^	“In the majority of cases of hearing loss associated with congenital viral infection, the damage to the auditory system is within the cochlea. It is likely that similar lesions account for the hearing deficit in children with congenital Zika virus infection, although histologic studies are needed to confirm this. However, a concomitant central origin cannot be discounted, and behavioral auditory evaluation might provide additional information”.
Leal et al. (2016); Brazil^8^	“It is still uncertain if the tissue damages caused by the intrauterine ZIKV infection are an expression of a direct effect of the virus itself or of an immune reaction from the host. Further histologic studies are necessary to determine the exact pathogenesis of the disease”.
Borja et al. (2017); Brazil^42^	“In this study, earlier absolute latencies were observed in children with microcephaly, with high-intensity stimuli for the investigation of the neurophysiological integrity of the brainstem. Considering the affinity of ZIKV for nerve tissue, it is possible that the neuroconduction of the acoustic stimulus in children exposed to the virus and with microcephaly is different from that of other children, even those exposed to other congenital infections. It is plausible to assume that the norms available in studies validated and used for the purpose of audiological diagnosis in term and preterm neonates do not apply to this population”.
	“… it should be considered that the neurological changes found in the imaging examinations imply the possibility of late hearing loss, cognitive and language alterations. Cognitive development is intrinsically linked to satisfactory relationships between sensory, perceptive, motor, linguistic, intellectual and psychological functions.”
Marques Abramov et al. (2018); Brazil^43^	“Our results about brainstem functional normality are challenging, considering the substantial disruption of brain development as well as evidence suggesting the action of ZV on progenitor cells, from the cell proliferation phase. Although the brainstem develops in parallel with the telencephalon in the early stages of embryogenesis, the development of the brainstem does not exhibit the same neuronal migration processes observed in the telencephalon, suggesting that the ZV primarily acts on specific mechanisms of cerebral cortex formation extending from the first to the third trimester.”
	“The functional organization of the brainstem, as observed in this study, indicates an adequate centripetal development process in children with microcephaly, with a neuronal and synaptic organization comparable to typical development, restricting the disorder produced by the ZV to more specific processes of CNS development, probably limited to the telencephalon.”
	“The increase of the deviation of the normality of latencies of wave I with the age at the time of examination suggests that the ZV infection leads to a progressive process in peripheral auditory nerve or sensorineural structures.”
Mittal et al. (2017); USA^46^	“Many infants with microcephaly exhibit SNHL without clear injury to the inner ear structures, possibly representing auditory impairment at the brainstem or cortical level. Another possibility could be inflammatory changes within the cochlea because the virus may have direct access to these structures via the cochlear aqueduct. But because of the association with microcephaly, some might question whether the HL in infants with ZIKV infection is central in origin due to brain malformation, rather than abnormality at the cochlear level”.
	“In summary, ZIKV exposure is associated with HL in infants and adults. Hearing loss can occur as a result of the damage to the inner ear or auditory nerve, by a direct viral effect or mediated by an autoimmune process as demonstrated in the case of other viral infections.”
Racicot et al. (2017); USA^47^	“Mutations affecting Diaph expression (Diaph 1 and 3) can cause microcephaly and hearing loss in humans and mice.”
	“Neurotropic viruses can significantly affect NPC (neuroprogenitor cell) functions, including cell fate decisions, proliferation, migration and survival.”
	“Most viruses express gene products that can functionally ‘hijack’ the Rho-Diaph system, sequestering these proteins for the purpose of modifying the actin cytoskeleton to promote viral entry, assembly and spread.”
	“We hypothesize that viral ‘repurposing’ of Diaphs disrupts normal host cellular functions, resulting in premature NPC differentiation or apoptosis, as mentioned previously. If viruses sequester Diaphs in NPCs, preventing them from executing their normal cellular functions, it follows that neurological phenotypes associated with these infections would be similar to those caused by genetic mutations of Diaphs.”
Leal et al. (2019); Brazil^52^	“In all studies, it seems clear that the auditory impairment is closely related to the presence of microcephaly and its severity.”
	“An issue not yet clarified is the topography of the lesion responsible for hearing loss produced by ZikV infection. None of these children had any anatomical abnormalities of the inner ear on imaging examinations, but it is well known that other congenital infections can cause deafness without any cochlear malformation; therefore, it is not possible to rule out a sensory impairment in these cases.”
	“…severe changes in the central nervous system of many of these newborns are a possible origin of the auditory impairment, as already demonstrated in other conditions that involve the auditory pathways.”
	“We are far from understanding if the lesions are produced directly by the virus itself or a result of an inflammatory tissue reaction. To date, no study has been addressed regarding a histologic analysis of the auditory sensory or neural structures.”

Three official documents were found, formalizing recommendations for auditory screening and follow-up in patients exposed to the ZIKV during the prenatal period: two interim guidelines published in the Morbidity and Mortality Weekly Report by the Centers for Disease Control and Prevention (CDC, Atlanta, GA, USA), with updates for the evaluation and management of children presenting possible congenital ZIKV infection^48^, ^49^ and a Manual by the Brazilian Ministry of Health (Protocol on Health Care and Response to the Occurrence of Microcephaly Related to Zika Virus Infection)^55^ (Table 7). In the last CDC Interim Guideline update,^48^ a change from the previous Interim Guideline^49^ was noted, ruling out the need for a new ABR between 4 and 6 months or a 9 month behavioral audiometry for children exposed to ZIKV during gestation if they presented a normal ABR performed during the first month of life.

**Table 7:** Recommendations on hearing screening and follow up for patients with congenital or acquired Zika virus infection.

Author, year, journal	Document title and publication type	Recommendations
Adebanjo et al., 2017^48^; MMWR – Morbidity and Mortality Weekly Report	Update: Interim Guidance for the Diagnosis, Evaluation, and Management of Infants with possible Congenital Zika Virus Infection – United States, October, 2017. Guideline article	Infants with clinical findings consistent with CZS or Infants without CZS who were born to mothers with laboratory evidence of possible maternal Zika virus infection during pregnancy aABR by age 1 month if the newborn hearing screen was passed using only OAE.
		Amendment to the previous recommendations of 2016: A diagnostic ABR at 4–6 months or behavioral audiology at age 9 months is no longer recommended if the initial hearing screen is passed by automated ABR because of absence of data suggesting delayed-onset hearing loss in congenital ZIKV infection.
Russell et al., 2016^49^; MMWR – Morbidity and Mortality Weekly Report	Update: Interim Guidance for the Diagnosis, Evaluation, and Management of Infants with possible Congenital Zika Virus Infection – United States, August 2016. Guideline article	Mothers with laboratory evidence of ZIKV with infants with no evidence of clinical abnormalities: Routine care, including PE, HC, weight/length and neurologic exam; Before discharge: hearing screen, postnatal head US; Infants testing positive for ZIKV: ABR and ophthalmology exam at age 2 wks and consider ABR at 4–6 months or behavioral audiology at age 9 months.
		Mothers with laboratory evidence of ZIKV, with infants with abnormalities consistent with CZS: Routine care, including PE, HC, weight/length and neurologic exam; Before discharge: hearing screen, ABR, postnatal head US, CBC, metabolic panel; LFTs; ophthalmologic exam, consider advanced neuroimaging; Consider transfer to hospital with subspecialty care; Infants testing negative: evaluate for other causes of congenital anomalies; further management as clinically indicated; Infants testing positive: thyroid screen by 2 wks and 3 mo; neurologic exam by 1 mo and 2 mo; ophthalmology exam by 3 mo and ABR by 4–6 mo; routine preventive health care including monitoring of feeding and growth; routine and congenital infection-specific anticipatory guidance; referral to specialists, including evaluation of other causes of congenital anomalies as needed.
		Mothers not tested for ZIKV or tested outside of appropriate Window^a^, with infants with no evidence of clinical abnormalities: Maternal ZIKV testing; consider ZIKV placental testing; Routine care, including PE, HC, weight/length and neurologic exam; Before discharge: hearing screen, postnatal head US; Infant ZIKV testing if evidence of ZIKV infection on maternal testing; Outpatient management for appropriate infant clinical exam and test results.
		Mothers not tested for ZIKV or tested outside of appropriate Window^a^, with infants with abnormalities consistent with CZS: Maternal ZIKV testing; consider ZIKV placental testing; Routine care, including PE, HC, weight/length and neurologic exam; Before discharge: hearing screen, ABR, postnatal head US, CBC, metabolic panel; LFTs; ophthalmologic exam, consider advanced neuroimaging; Consider transfer to hospital with subspecialty care; Infants testing negative: evaluate for other causes of congenital anomalies; further management as clinically indicated; Infants testing positive: refer to outpatient management for infant with abnormalities consistent with congenital Zika syndrome.
Brasil. Ministério da Saúde. Scretaria de Atenção à Saúde, 2015.^55^ Available from: www.saude.gov.br/svs	Protocol for Health Care and Response to the Occurrence of Microcephaly related to Zika virus Infection. Manual of Health Care	Newborn with microcephaly: Hearing screening test (OAE) with 24–48 h of life and ABR ideally in the maternity. If there is no equipment for ABR, send the patient to the nearest referral service (Specialized Center for Rehabilitation with hearing modality or High Complexity Auditory Rehabilitation Center), until the first month of life. In case of failure, retest must be performed within 30 days, preferably at the same place as the previous test. In case of retest failure, the child should be immediately referred for otorhinolaryngological and audiological diagnostic evaluation. Neonatal hearing screening test should not be performed in children with ear malformations (even if unilateral). These patients should be sent directly to a referral service for otorhinolaryngological and audiological diagnosis, according to the Neonatal Hearing Screening Guidelines. If hearing loss is diagnosed, the child should be sent for rehabilitation in reference service in auditory rehabilitation: Specialized Center in Rehabilitation with hearing modality or High Complexity. Auditory Rehabilitation Center. Microcephaly is a risk indicator for hearing loss.
Leal et al., 2016^9^; MMWR – Morbidity and Mortality Weekly Report	Hearing Loss in Infants with Microcephaly and Evidence of Congenital Zika Virus Infection – Brazil, November 2015–May 2016. Case series	To elucidate the full spectrum of hearing loss in infants with congenital Zika virus infection, testing and follow-up of all children born to women who had Zika virus infection during pregnancy, including infants with no apparent anomalies at birth, is needed.
		Sensorineural hearing loss should be considered part of the spectrum of clinical findings associated with congenital Zika virus infection, and congenital Zika virus infection should be considered a risk factor for hearing loss in auditory screening programs.
		Children with evidence of congenital Zika virus infection who have normal initial screening tests should receive regular follow-up, because onset of hearing loss could be delayed and the loss could be progressive.
Leal et al., 2016^8^; Brazilian Journal of Otorhinolaryngology	Sensorineural hearing loss in a case of congenital Zika virus. Case report	In hearing assessments protocols for neonates, mother's infection by Zika virus should be included among the risk factors for hearing loss.
Borja et al., 2017^42^; Revista de Ciências médicas e Biológicas	Hearing screening in children exposed to Zika virus during pregnancy. Case series	The health services that provide care to this population (children exposed to Zika virus during pregnancy) should make parents or caregivers aware of the need to continue monitoring of hearing development up to 24 months of age, even if the child has passed the screening tests, understanding that late hearing loss may occur, as well as auditory development may present important delays that may compromise the development of language.
Satterfield-Nash et al., 2017^10^; MMWR – Morbidity and Mortality Weekly Report	Health and Development at Age 19–24 Months of 19 Children Who Were Born with Microcephaly and Laboratory Evidence of Congenital Zika Virus Infection During the 2015 Zika Virus Outbreak – Brazil, 2017. Case series^b^	These findings allow for anticipation of medical and social service needs of affected children and their families, including early intervention services, and planning for resources to support these families in health care and community settings in Brazil, the United States, and other countries.
		Children with disabilities related to congenital Zika virus infection will need multidisciplinary care from various pediatric subspecialists (10).
		Long-term follow-up and measurement of developmental progression of children affected by Zika virus can inform intervention services and sub-specialties needed to provide optimal care for these children.
Silva et al., 2017.^11^ Available from: www.conbracis.com.br	Hearing screening in children exposed to zika virus. Case series^c^	It is recommended that infants with microcephaly, exposed to the Zika virus, be screened with a-BAEP, since exposure to the Zika virus has been described as a risk indicator for hearing loss (RIHL) (Ministry of Health, 2016). The justification for performing this test in children with RIHL is the higher prevalence of non-identifiable retrocochlear hearing loss through otoacoustic emissions (MINISTRY OF HEALTH, 2012).
Mittal et al., 2017^46^; JAMA Otolaryngology – Head & Neck Surgery	A Possible Association Between Hearing Loss and Zika Virus Infection. Opinion article	Otolaryngologists should monitor ZIKV exposed infants without hearing impairment at birth because they may develop HL at later stages of life.
		The early diagnosis and detection of HL in ZIKV-exposed infants will improve auditory rehabilitation, leading to improved long-term developmental outcomes.
Fandiño-Cárdenas et al., 2018^44^; Journal of tropical Pediatrics	Zika Virus Infection during Pregnancy and Sensorineural Hearing Loss among Children at 3 and 24 Months Post-Partum. Case series	Hearing loss because of congenital ZIKV can be sensorineural, neural, conductive, alone or mixed. Therefore, a complete hearing assessment, including aABR and DPOAE, should be performed on all ZIKA-infected patients, thus ruling out auditory neuropathy syndrome and sensorineural hearing loss.
		Regardless if microcephaly is present, every neonate born with suspicious of gestational or congenital Zika infection needs to be tested at birth and in a follow-up with a complete audiology assessment, given the potential impairment of hearing over time, as occurs in CMV.
		The nature of the hearing loss in ZIKV infection may be progressive, and a follow-up hearing test must be conducted at least during the first 5 years o life.
Martins et al., 2017^6^; Audiology Communication Research	Otological findings in patients following infection with Zika virus: case report. Case series	In view of the rapid spread of ZIKV in Brazil, it is suggested that patients should monitor their auditory health following ZIKV infection, since even though those patients may not report any alterations in their hearing accuracy; it is possible that the central auditory system could be affected.
Vinhaes et al., 2017^7^; Clinical Infectious Disease	Transient Hearing Loss in Adults Associated With Zika Virus Infection. Case series	Further investigation might also highlight other possible rare events such as permanent hearing loss, facilitating the possible recommendation of audiometry examinations in adults during ZIKV outbreaks.
Peloggia et al., 2018^53^; International Journal of Gynecology and Obstetrics	Zika virus exposure in pregnancy and its association with newborn visual anomalies and hearing loss. Narrative review	Hearing examination of infants with suspected CZS infection, even in the absence of microcephaly, is essential, because the associated impairments might be underestimated if microcephaly continues to be the only inclusion criterion during the screening of this group of infants.
Leite et al., 2018^13^; Epidemiologia e Serviços de Saúde	Hearing Screening in children with Congenital Zika Virus Syndrome in Fortaleza, Ceará, Brazil, 2016. Case series	The inclusion of the tympanometry in the hearing screening before the referral to the ABR is suggested.
		The hearing screening should be performed in children with CZS right after birth and the referral to the clinical and audiological diagnosis occur only in those who fail the screening, in early age.
		The a-ABR should be included in the hearing screening recommendation for children with CZS.
Lage et al., 2019^14^; International Journal of Environmental Research and Public Health	Clinical, Neuroimaging, and Neurophysiological Findings in Children with Microcephaly Related to Congenital Zika Virus Infection. Case series	Children with microcephaly related to CZS need regular follow-ups, even the ones with normal initial screening tests, because hearing loss, like in other congenital viral infections, can be delayed and progressive.
Leal et al., 2019^52^; Topics in Magnetic Resonance Imaging	Hearing Loss From Congenital Zika Virus Infection. Narrative Review	As congenital ZIKV infection should be considered a risk factor for hearing loss, neonatal hearing screening with auditory-evoked potential is recommended, maintaining clinical follow-up, even for those who pass the screening, with at least 1 audiological evaluation in the period between 24 and 30 months of age, as recommended for children who present risk factors for hearing loss.
Calle-Giraldo et al., 2019^16^; The Pediatric Infectious Disease Journal	Outcomes of Congenital Zika Virus Infection During an Outbreak in Valle del Cauca, Colombia. Case series	Infants exposed during pregnancy should receive close neurologic, ophthalmologic and audiologic monitoring, even in the absence of microcephaly.

a-ABR, automated Auditory Brainstem Response; CZS, Congenital Zika Virus Syndrome; HC, Head (occipitofrontal) Circumference; OAE, Otoacoustic Emissions; PE, Physical Exam; ZIKV, Zika Virus.

a Mothers should be tested by rRT-PCR within 2 weeks of exposure or symptom onset, or by IgM within 2–12 weeks of exposure or symptom onset. Because of the decline in IgM antibody titers and viral RNA levels over time, negative maternal testing 12 weeks after exposure does not rule out maternal infection.

b Sub-sample from Zodiac (Zika Outcomes and Development in Infants and Children) case–control study.

c Presented at the II Brazilian Congress of Health Sciences.

In 11 case series type articles,^6^, ^7^, ^8^, ^9^, ^10^, ^11^, ^13^, ^14^, ^16^, ^42^, ^44^ one opinion piece^46^ and two reviews,^52^, ^53^ the authors also suggest recommendations for screening and auditory monitoring concerning prenatal exposure to the ZIKV. Most studies recommend that this condition be considered as a risk indicator for hearing loss and, therefore, that children should be screened by OAE and a-ABR. They also highlight the need for regular follow-up regarding hearing development, due to the possibility of late hearing loss^9^, ^10^, ^42^, ^46^ (Table 7).

In two studies reporting cases of auditory impairment in ZIKV-acquired infections, the authors suggest auditory monitoring in these cases^6^ and the need for further investigations to promote this recommendation during ZIKV outbreaks^7^ (Table 7).

## Discussion

Despite the involvement of several research groups in the study of ZIKV infection, this review found only 27 records that specifically contained one or more of the three proposed outcomes related to congenital or acquired ZIKV infection: auditory, functional and/or morphological manifestations, hypotheses concerning the pathogenesis of auditory system alterations and recommendations regarding the screening or follow-up of individuals with hearing disorders related to ZIKV infection.

Only three case reports were published describing auditory changes related to acquired ZIKV infection in adults, totaling six participants.^5^, ^6^, ^7^ The predominantly sensorineural and transient nature of auditory alterations raises the hypothesis of auditory organ cochlear or neural involvement. An association between flavivirus infection and hearing loss was suggested in two other reports concerning DENV infection cases. However, vascular impairment could not be ruled out in one of the reports,^56^ describing an adult with hemorrhagic dengue, who presented deep and bilateral sensorineural hearing loss after 5 days of symptoms. In another report^57^ of a child presenting vertical infection by DENV and a clinical condition of hemorrhagic shock who presented alteration during hearing screening, other risk indicators for hearing impairment were also present, such as low birth weight, use of ototoxic medications and permanence in the Neonatal ICU. These findings do not allow us to state that acquired ZIKV or DENV infections cause auditory impairment. However, unlike DENV cases, which comprised other risk factors for hearing loss, in the three ZIKV infection cases, the absence of other events that justify auditory pathway involvement, alongside the strong tropism of the virus by the nervous system, reinforce the hypothesis that damage to these pathways was due to ZIKV infection.

In studies that assessed auditory impairment in prenatal exposure to the ZIKV, it is noteworthy that only about two-thirds of all children underwent hearing assessment, with the majority of them being at an age far above one month as recommended by national and international protocols.^48^, ^55^ Hearing assessments were carried out predominantly by means of OAE and/or ABR to obtain auditory thresholds. The large variation in the frequency of alterations found during auditory evaluations may be partially explained by the great heterogeneity of the study population, methods of hearing assessment and study designs.

The findings favor the hypothesis of an auditory risk of probable peripheral origin, since the predominant alterations were OAE failure and auditory threshold changes assessed by the ABR. On the other hand, conductive alterations, suggested by children who failed the first OAE screening and presented a normal electrophysiological response in the ABR click assessment,^42^ sensorineural alterations, suggested by children who failed both tests, or retrocochlear alterations, in the cases which passed the T-OAE and failed the ABR,^11^ have also been described in various studies. Some children presented other risk factors for hearing loss, and very few studies performed a broad auditory evaluation, including FS-ABR, behavioral auditory assessment^8^ or tympanometry.^13^, ^44^ In addition, losses of a central origin could not be ruled out.

In a small sample of children who presented with late microcephaly^51^ and, therefore, less severe neurological damage than microcephaly at birth, the absence of alterations in the auditory evaluations performed by a-ABR and FS-ABR was observed. Similarly, in a transversal study of hearing screening including children congenitally exposed to ZIKV with or without microcephaly, no alterations suggesting sensorineural hearing loss were observed.^13^ These results raise suggest a possible relation between the degree of neurological damage and auditory impairment, also emphasized by Leal et al.^52^ in a review piece. On the other hand, Marques Abramov et al.^43^ concluded, in an evaluation of the conduction of the auditory pathways up to the brainstem in patients with prenatal exposure to ZIKV, that the physiology of auditory pathways of the brainstem is not affected by ZIKV congenital infection, even when it occurs during the first trimester, and that there is no direct correlation between the degree of microcephaly and auditory pathway function in the brainstem. Finally, the need for long-term follow-up remains controversial. The presence of auditory alterations in late evaluations, at 19–24 months of life, in 68.4% of microcephalic children at birth, with laboratory evidence of congenital infection by ZIKV, reinforces the need for late follow-up. These evaluations, however, were restricted to the Hammersmith Infant Neurologic Exam (HINE) – a response to sound stimulation with a rattle or bell in a small sample of children, where it was not possible to identify the type of auditory alteration, central or peripheral, sensorineural or conductive.^10^ On the other hand, in a recent review Leal et al.^52^ mentioned a not yet published study that found an incidence of 4.3% of hearing loss in newborns with congenital ZIKV infection and a normal auditory follow-up of those children after 18 months, contradicting the possibility of a progressive or late onset hearing loss. In the only experimental study in no-human mammals,^54^ it is noteworthy that in several animals the deficits improved in subsequent measurements, suggesting a transitory hearing loss, as observed in the acquired human infection,^5^, ^6^, ^7^ but also contradicting the hypothesis of a progressive hearing loss.^9^, ^10^, ^43^, ^52^

Some of the analyzed studies make suggestions concerning the pathogenesis of auditory involvement by the ZIKV, generally based on what is observed in other viral infections (Table 5). In the animal study previously mentioned, the microscopic analysis detected viral antigens in the cochlea, but no changes in the number of hair cells were observed, leading to the postulation that the hearing loss associated to ZIKV infection does not seem to involve damaged hair cells.

It is not known if the tissue lesion is due to the direct effect of the virus or the host's immune reaction,^7^, ^8^ where it may involve only the cochlea or may originate from the central nervous system, especially in cases of CNS malformations^9^, ^46^ or if the neuroconduction of the acoustic stimulus is involved, both in prenatal^42^ and postnatal exposure,^6^ which are issues that require clarification. There are indications of preservation of functional organization in the brainstem of microcephalic children exposed to the ZIKV during pregnancy, suggesting that the disorders caused by the ZIKV are restricted to more central regions.^43^ More importantly, there is evidence of a possible progressive damage to the peripheral regions of the auditory nerve or to sensorineural structures, as suggested by Marques Abramov et al.^43^ from the finding of increased I wave latency in the ABR with age. This progressive damage could be responsible for late hearing loss.

The hypothesis raised by Racicot et al.,^47^ suggesting that the ZIKV causes sequestration and redirection of proteins (Diaphanous-related formins – Diaphs) in progenitor neural cells, determining microcephaly or hearing loss in humans, similar to what is noted in individuals presenting mutations in the genes responsible for the expression of these proteins, opens a promising field to be explored in future research.

Regarding the guidelines for screening and auditory follow-up in individuals exposed to ZIKV, controversies regarding the need to repeat subsequent auditory evaluations in the presence of a normal ABR in the neonatal period were observed herein. Although the authors of the last CDC Interim Guideline^48^ justify the lack of need for reevaluation based on the lack of data suggesting late hearing loss in congenital ZIKV infection, the opinion of the authors of this review is that the evidence on the absence of late impairment are still insufficient to support these recommendations. Long-term follow-up studies of children exposed to ZIKV during gestation are necessary for the establishment of evidence-based recommendations.

This review presents some limitations. Most of the studies are retrospective reports or case series, assessing small sample sizes and carrying out cross-sectional auditory evaluations, which makes it difficult to establish causal links and prognostic projections to generate enough evidence to propose well-founded protocols. Moreover, the included studies were very heterogeneous in many aspects, which makes it difficult to compare and synthesize the data collected. Additionally, not all studies focused on hearing assessments, restricted to only neonatal screening and providing minimal details on the performed tests or the obtained results. Many studies limited the hearing evaluation to small sub-samples of patients presenting the more severe viral infection spectrum. Therefore, it was not possible to estimate the overall frequency of altered hearing exams in the subgroup of children with confirmed congenital ZIKV infection, since laboratory confirmation was not specified in the children who performed auditory evaluations. However, three studies that included only children with confirmed ZIKV infection allowed the frequency estimation of auditory alterations in this subset of children. We opted to analyze the frequency of auditory alterations in the microcephaly group, to determine if a worst spectrum of clinical manifestations could be related with an increased frequency of hearing alterations. Unfortunately, the relative frequency of altered a-ABR, which was the most frequently performed test and more specific than de OAE, could not be estimated in children with microcephaly, because of missing data. Finally, the absence of studies evaluating auditory organ histopathology in human ZIKV infection restricts knowledge of the pathogenesis of auditory impairment to hypotheses and theories, limiting the effectiveness of prevention, monitoring and therapeutic management actions. However, in the case of a recent and large epidemic, whose tragic legacy has only recently been recognized, these early studies become a valuable source of data.

## Conclusions

Evidence for the involvement of the auditory pathways in congenital or acquired infection by ZIKV is still scarce. The data available to date do not allow the knowledge of the entire spectrum of auditory organ involvement by ZIKV, nor do they confirm the causal association between this involvement and the virus infection, nor they rule out progressive hearing impairment. Especially with regard to individuals with central nervous system malformations, data are still missing to confirm hearing loss of central origin. Multidisciplinary monitoring of all children exposed to ZIKV during pregnancy should improve, with earlier hearing assessment and follow-up. Future research resulting from the long-term follow-up of children presenting the full spectrum of ZIKV involvement, as well as necropsies of stillborn hearing organs and more studies with animal models may provide answers to these yet unanswered questions.

## Conflicts of interest

The authors declare no conflicts of interest.

## Figures and Tables

**Table:** 

Database	Search strategy	Results (*n*)
PUBMED	(((Zika[Title/Abstract] OR ZIKV[Title/Abstract]))) AND ((Acoustic OR Audiometry OR Tympanometry OR Auditory OR “Evoked Potentials” OR Psychoacoustics OR “Evoked Response” OR P300 OR ABR OR BERA) OR (Hearing OR Hypoacusis OR Deafness OR Audition OR Dysacusis OR cochlear OR retrocochlear))	32
SCOPUS	(TITLE-ABS-KEY (zika OR zikv)) AND ((TITLE-ASB-KEY (acoustic OR audiometry OR tympanometry OR auditory OR “Evoked Potentials” OR psychoacoustics OR “Evoked Response” OR p300 OR abr OR bera)) OR (TITLE-ABS-KEY (hearing OR hypoacusis OR deafness OR audition OR dysacusis OR cochlear OR retrocochlear)))	58
SCIELO	(Zika OR ZIKV) AND (Acoustic OR Audiometry OR Tympanometry OR Auditory OR “Evoked Potentials” OR Psychoacoustics OR “Evoked Response” OR P300 OR ABR OR BERA OR Hearing OR Hypoacusis OR Deafness OR Audition OR Dysacusis OR cochlear OR retrocochlear)	15
LILACS	(Zika OR ZIKV) AND (Acoustic OR Audiometry OR Tympanometry OR Auditory OR “Evoked Potentials” OR Psychoacoustics OR “Evoked Response” OR P300 OR ABR OR BERA OR Hearing OR Hypoacusis OR Deafness OR Audition OR Dysacusis OR cochlear OR retrocochlear)	21
Web of Science	(TITLE-ABS-KEY (zika OR zikv)) AND ((TITLE-ASB-KEY (acoustic OR audiometry OR tympanometry OR auditory OR “Evoked Potentials” OR psychoacoustics OR “Evoked Response” OR p300 OR abr OR bera)) OR (TITLE-ABS-KEY (hearing OR hypoacusis OR deafness OR audition OR dysacusis OR cochlear OR retrocochlear)))	33
GOOGLE/GOOGLE Scholar	Zika AND hearing	5

**Table:** 

Title	1	The words “case report” (or “case study”) should be in the title along with phenomenon of greatest interest (e.g., symptom, diagnosis, test, intervention)
Keywords	2	The key elements of this case in 2–5 words.
Abstract	3a	a) Introduction – What does this case add?
	3b	b) Case presentation: The main symptoms of the patient; The main clinical findings; The main diagnoses and interventions; The main outcomes
	3c	c) Conclusion – What were the main “take-away” lessons from this case?
Introduction	4	Brief background summary of the case referencing the relevant medical literature.
Patient information	5a	Demographic information of the patient (age, gender, ethnicity, occupation)
	5b	Main symptoms of the patient (his or her chief complaints)
	5c	Medical, family, and psychosocial history including diet, lifestyle, and genetic information whenever possible and details about relevant comorbidities and past interventions and their outcomes
Clinical findings	6	Describe the relevant Physical Examination (PE) findings
Timeline	7	Depict important dates and times in the case (Table or Figure)
Diagnostic assessment	8a	Diagnostic methods (e.g., PE, laboratory testing, imaging, questionnaires)
	8b	Diagnostic challenges (e.g., financial, language/cultural)
	8c	Diagnostic reasoning including other diagnoses considered
	8d	Prognostic characteristics (e.g., staging) where applicable
Therapeutic interventions	9a	Types of intervention (e.g., pharmacologic, surgical, preventive, self-care)
	9b	Administration (e.g., dosage, strength, duration)
	9c	Changes in intervention (with rationale)
Follow-up and outcomes	10a	Clinician and patient-assessed outcomes
	10b	Important follow-up test results (positive or negative)
	10c	Intervention adherence and tolerability (and how this was assessed)
	10d	Adverse and unanticipated events
Discussion	11a	Strengths and limitations of the management of this case
	11b	Relevant medical literature
	11c	Rationale for conclusions (including assessments of cause and effect)
	11d	Main “take-away” lessons of this case report
Patient perspective	12	The patient should share their perspective or experience whenever possible
Informed consent	13	Did the patient give informed consent? Please provide if requested

**Table:** 

Title	1	Are words related to the main issue (hearing) in the title?
Keywords	2	2 to 5 keywords to identify areas of interest in the case report
Abstract	3a	Introduction – One statement contextualizing and justifying the study theme?
	3b	Does the abstract present objectives, methods, results and conclusions?
Introduction	4	One or two paragraphs contextualizing and justifying the theme referencing the relevant medical literature
Objectives	5	Was the study question or objective clearly stated?
Participants	6a	Was the study population clearly specified and defined?
	6b	Was a sample size justification, power description, or variance and effect estimates provided?
	6c	Were all the subjects selected or recruited from the same or similar populations (including the same time period)? Were inclusion and exclusion criteria for being in the study prespecified and applied uniformly to all participants?
	6d	Were the definitions, inclusion and exclusion criteria, algorithms or processes used to identify or select cases and controls valid, reliable, and implemented consistently across all study participants?
	6e	Were the cases clearly defined and differentiated from controls?
	6f	If less than 100 percent of eligible cases and/or controls were selected for the study, were the cases and/or controls randomly selected from those eligible?
	6g	Was there use of concurrent controls?
Evaluation of exposure	7a	Were the investigators able to confirm that the exposure/risk occurred prior to the development of the condition or event that defined a participant as a case?
	7b	Were the measures of exposure/risk clearly defined, valid, reliable, and implemented consistently (including the same time period) across all study participants?
	7c	Were the assessors of exposure/risk blinded to the case or control status of participants?
Results	10a	Were the results well-described? (Does it present adequate descriptive statistics?)
	10b	Were there comparisons between subgroups, association measures with adequate statistics (precision measures)?
	10c	Were key potential confounding variables measured and adjusted statistically in the analyses? If matching was used, did the investigators account for matching during study analysis?
Discussion	11a	Strengths and limitations of the management of this case
	11b	Discussion of relevant medical literature
	11c	Rationale for conclusions (including assessments of cause and effect)
Conclusion	12	What were the main “take-away” lessons from this case?
Informed consent	13	Did the patient give informed consent?
